# COVID-19 Pneumonia and Lung Cancer: A Challenge for the Radiologist Review of the Main Radiological Features, Differential Diagnosis and Overlapping Pathologies

**DOI:** 10.3390/tomography8010041

**Published:** 2022-02-11

**Authors:** Alessia Guarnera, Elena Santini, Pierfrancesco Podda

**Affiliations:** 1 Radiology Department, San Giovanni Addolorata Hospital, 00184 Rome, Italy; elensantini@tiscali.it (E.S.); pfpodda@hsangiovanni.roma.it (P.P.); 2 Neuroradiology Unit, NESMOS Department, Sant’Andrea Hospital, La Sapienza University, 00189 Rome, Italy

**Keywords:** COVID-19, omicron, variants, WHO, 2021, lung cancer, cancer, ground glass, SARS-CoV-2, pneumonia, crazy paving, artificial intelligence

## Abstract

The COVID-19 pneumonia pandemic represents the most severe health emergency of the 21st century and has been monopolizing health systems’ economic and human resources world-wide. Cancer patients have been suffering from the health systems’ COVID-19 priority management with evidence of late diagnosis leading to patients’ poor prognosis and late medical treatment. The radiologist plays a pivotal role as CT represents a non-invasive radiological technique which may help to identify possible overlap and differential diagnosis between COVID-19 pneumonia and lung cancer, which represents the most frequent cancer histology in COVID-19 patients. Our aims are: to present the main CT features of COVID-19 pneumonia; to provide the main differential diagnosis with lung cancer, chemotherapy-, immunotherapy-, and radiotherapy-induced lung disease; and to suggest practical tips and key radiological elements to identify possible overlap between COVID-19 pneumonia and lung cancer. Despite similarities or overlapping findings, the combination of clinics and some specific radiological findings, which are also identified by comparison with previous and follow-up CT scans, may guide differential diagnosis. It is crucial to search for typical COVID-19 pneumonia phase progression and typical radiological features on HRTC. The evidence of atypical findings such as lymphadenopathies and mediastinal and vessel invasion, as well as the absence of response to therapy, should arouse the suspicion of lung cancer and require contrast administration. Ground-glass areas and/or consolidations bound to radiotherapy fields or pneumonitis arising during and after oncological therapy should always arouse the suspicion of radiation-induced lung disease and chemo/immunotherapy-induced lung disease. The radiological elements we suggest for COVID-19 and lung cancer differential diagnosis may be used to develop AI protocols to guarantee an early and proper diagnosis and treatment to improve patients’ quality of life and life expectancy.

## 1. Introduction

COVID-19 pneumonia represents the most severe pandemic of the 21st century with high interpersonal transmission through virus inhalation [1] and with continuous incoming variants, the last being the omicron, which has led health systems to further focus their financial and human resources on its diagnosis and treatment [2,3], while governments all around the world have been fighting the pandemics with lockdowns, social distancing, and vaccination [4,5].

The scientific community has focused its efforts on COVID-19 diagnosis and treatment, showing great interest in new scientific fields such as artificial intelligence (AI). Artificial intelligence has been suggested for early identification of COVID-19 clusters, disease monitoring, and mortality risk prediction with good accuracy [6,7,8].

Non-COVID diseases, and in particular oncological diseases, suffered from health systems’ COVID-19 priority management with evidence of late diagnosis leading to patients’ poor prognosis and late medical treatment [9,10].

Oncological patients are more prone to be infected by COVID-19 and to present severe forms of pneumonia and complications due to immunosuppression caused by cancer and chemotherapy-immunosuppressive therapies and because they regularly visit hospitals for restaging and treatment [11,12,13,14]. Sometimes a COVID-19 pneumonia diagnosis is made on CT scans in cancer patients with no specific COVID-19 symptoms [15] and differential diagnosis between COVID-19 pneumonia and lung cancer may be complicated [16].

This dire scenario highlights the need to investigate possible overlap and differential diagnosis between COVID-19 pneumonia and lung tumors, which is the most frequent cancer histology in COVID-19 patients [17].

The radiologist plays a crucial role as CT represents a non-invasive radiological technique that may suggest COVID-19 diagnosis or possible differential diagnoses.

Our primary aims are to present the main CT features of COVID-19 pneumonia and to provide the main differential diagnosis with lung cancer according to the 2021 WHO classification [18], and with chemotherapy-, immunotherapy-, and radiotherapy-induced lung diseases. The secondary aim is to provide practical tips and key radiological elements to identify possible overlap between COVID-19 pneumonia and lung cancer.

## 2. COVID-19 Pneumonia

COVID-19 infection is contracted through the inhalation of the SARS-CoV-2, which links to the receptor of ACE II of alveolar and endothelium cells [1,19]. Secondary inflammation and coagulation system activation has been proved to contribute to severe complications of COVID-19 pneumonia which may be appreciated in all the organs and systems of human body from ARDS of the lung, to vascular thrombosis, to pulmonary embolism, and to neuro-COVID [20,21,22].

COVID-19 patients’ clinical presentation ranges from completely asymptomatic to highly symptomatic and symptoms may vary from very typical, such as ageusia and anosmia, to non-specific symptoms such as cough and mild fever [1].

Therefore, COVID-19 asymptomatic patients may be accidentally identified through radiological exams performed for other reasons. Among these reasons, serial chest CT exams are performed in oncological patients and, in particular, in lung cancer patients. Despite its availability, cost-effectiveness, and easy decontamination, chest X-rays are insensitive in early and mild COVID-19 pneumonia and not sufficient in the staging and restaging of lung cancer patients. On the other hand, CT represents the optimal exam for early diagnosis and disease progression monitoring [23,24,25].

**Figure 1:**
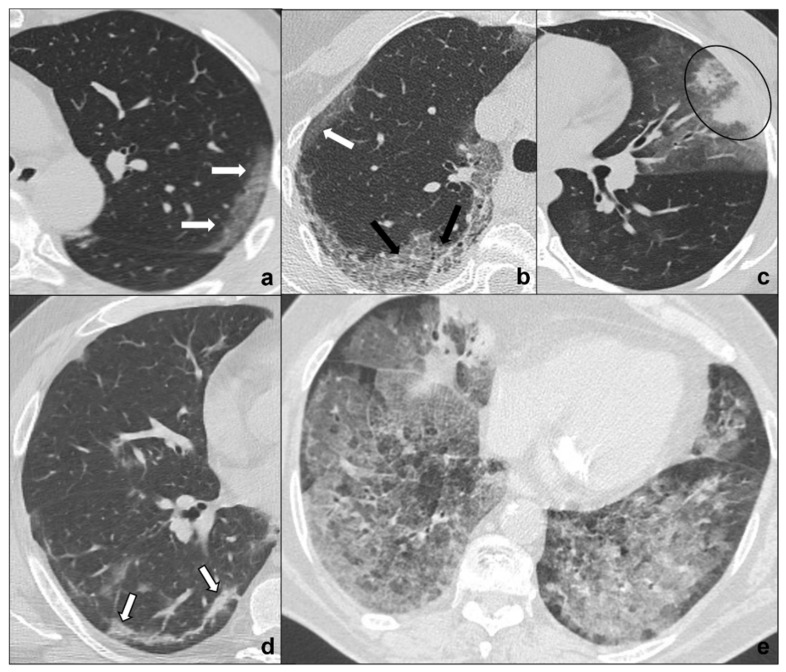
(**a**–**e**) HRTCs showing the four different stages of COVID-19 pneumonia (**a**–**d**) and ARDS as a possible complication I. In (**a**), the first stage is characterized by subpleural ground-glass opacities (white arrows in (**a**,**b**)) which co-exist with extensive subpleural crazy paving pattern in stage 2 (black arrows in (**b**); in (**c**), consolidations (black circle) are typical of phase 3 and may be surrounded by ground-glass opacities; in (**d**) parenchymal bands (black-bordered white arrows in (**d**) are characteristic of the absorption phase); in (**e**), confluent ground glass opacities with dependent foci of consolidations represent a dire complication of COVID-19 pneumonia: acute respiratory distress syndrome. Lungs: mean window with 1500 HU; mean window level −600. Mediastinum: mean window with 350 HU; mean window level 50 HU.

COVID-19 pneumonia progression typically encompasses four stages that may overlap each other [26] (Figure 1, Table 1):

1. Early phase or phase one (between day 0 and day 4) is characterized by bilateral and diffuse subpleural ground-glass opacities (Figure 1a);
2. Progressive phase or phase two (between day 5 and 8) shows extensive subpleural crazy paving areas co-existing with ground-glass opacities. Small consolidative foci may be present (Figure 1b);
3. Peak phase or phase three (between day 9 and 13) is defined by subpleural consolidation with peripheral ground glass and/or crazy paving opacities (halo sign) (Figure 1c);
4. Absorption phase or phase four (14 days and beyond) is the final stage in which parenchymal bands and ground-glass opacities represent a process of parenchymal repair and reorganization mediated by organizing pneumonia [27] (Figure 1d).

**Figure 2:**
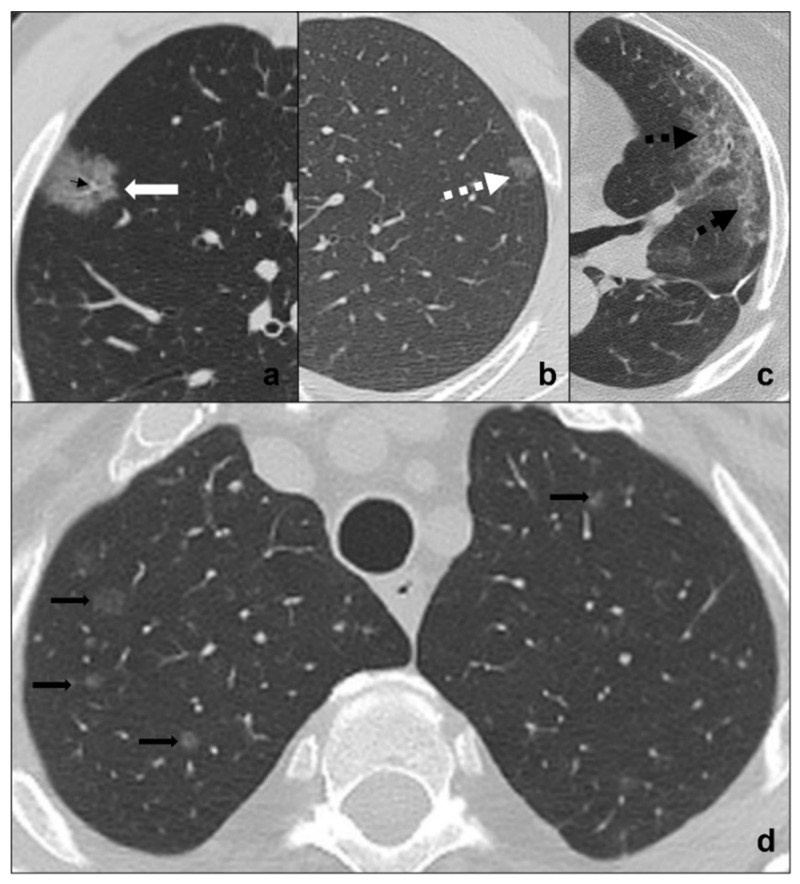
(**a**–**d**) HRTCs showing differential diagnoses of COVID-19 pneumonia stage 1/ground-glass opacities. COVID-19 phase 1 is characterized by subpleural ground-glass areas (white arrow in (**a**)) with ectasis vessels within (black arrow in (**a**)). Its main differentials are represented by adenocarcinoma in situ, presenting as a focal area of ground-glass opacity (dotted black arrow in (**b**)); radiation pneumonitis (acute radiation-induced lung disease) presenting with ground-glass opacities (dotted black arrows in (**c**)) in the field of radiation; and ground-glass metastases (black arrows in (**d**)) which appear as multiple focal ground-glass opacities with a random distribution. Lungs: mean window with 1500 HU; mean window level −600. Mediastinum: mean window with 350 HU; mean window level 50 HU.

Additional findings are [26,28]:

5. bilateral and subpleural distribution of lesions, in particular in the inferior lobes;
6. peripheral pulmonary vessels ectasis, especially within ground-glass/crazy paving opacities (Figure 2a);
7. atoll sign or reverse halo sign consisting in an area of ground-glass surrounded by a consolidation halo [28,29].

Rare findings that should raise the suspicion of a differential diagnosis are lymphadenopathies, pulmonary nodules, cavitations, and pleural effusions [26,28].

Patients may experience a sudden worsening of clinical conditions presenting with tachypnea, air hunger, and wheezing indicating a progression to a severe COVID-19 pneumonia complication: ARDS (acute respiratory distress syndrome) [30]. Chest CT shows patchy ground glass areas which progress to confluent opacities coexisting with dependent consolidations with a typical antero-posterior gradient [31].

## 3. COVID-19 and Lung Cancer: Differential Diagnosis

The 2021 WHO classification of lung tumors endorsed the basic principles of the 2015 WHO classification by suggesting an optimal pipeline to approach lung cancer starting from the morphological appearance supported by immunohistochemistry and molecular techniques, with a major emphasis on molecular pathology [18,33].

The multidisciplinary team, through the evaluation of clinics, anamnesis, laboratory imaging, and histopathological findings, guarantees a proper diagnosis and elaborates an optimal treatment. The role of the radiologist is to suggest a lung cancer diagnosis and possible differential diagnoses, especially in the COVID-19 era in which atypical presentations and overlapping pathologies are frequent findings [13].

Imaging provides a morphological approach to lung pathology. Differential diagnosis on CT is made by evaluating radiological signs, lesion distribution and timing, and contrast enhancement.

We will focus on the most frequent cancer- and treatment-related differential diagnoses by using a morphological approach based on the radiological findings identified in the four phases of COVID-19 pneumonia [26] (Table 1).

### 3.1. Ground-Glass

#### 3.1.1. Single Pure Ground-Glass Lesion

##### Adenocarcinoma Precursor Glandular Lesions

AIS (adenocarcinoma in situ) and AAH (atypical adenomatous hyperplasia) are non-invasive pure lepidic-growing preinvasive lesions that may be seen as focal ground glass opacities on CT scan. They represent a morphologic continuum and dimensional criteria may be useful to differentiate them, AAH likely being ≤5 mm, AIS generally being bigger and ≤3 cm [34,35,36] (Figure 2b).

Radiological features for differential diagnosis between precursor glandular lesions and COVID-19 pneumonia (Figure 2a,b) [34,35,36]):

- generally single and focal ground-glass opacity;
- distribution is random and may be centro-parenchymal or subpleural;
- asymptomatic and resistant to antibiotic and anti-inflammatory treatment;
- tendency to grow and/or evolve to malignancy with different timing with respect to COVID-19 pneumonia.

#### 3.1.2. Multiple Pure Ground-Glass Lesion

##### Metastases

Ground-glass metastases are less frequent than solid metastases and present as multiple focal areas of ground-glass opacities with a random distribution. Although patients commonly present with an already-known primitive tumor diagnosis and do not present acute symptoms, ground-glass metastases may be the presentation of cancer [37,38,39] (Figure 2d).

Radiological features for differential diagnosis between ground-glass metastases and COVID-19 pneumonia [37] (Figure 2a,d):

1. evidence of coexisting solid/subsolid lung cancer or systemic known cancer;
2. bilateral involvement with a random distribution in case of hematogenous spread or with spread thought airspace (STAS);
3. ground-glass opacities do not follow COVID-19 phases and are generally asymptomatic in early phases;
4. growing despite antibiotics and anti-inflammatory therapies;
5. pleural effusions;
6. mediastinal lymphadenomegalies.

### 3.2. Crazy Paving

#### Lymphangitic Carcinomatosis

**Figure 3:**
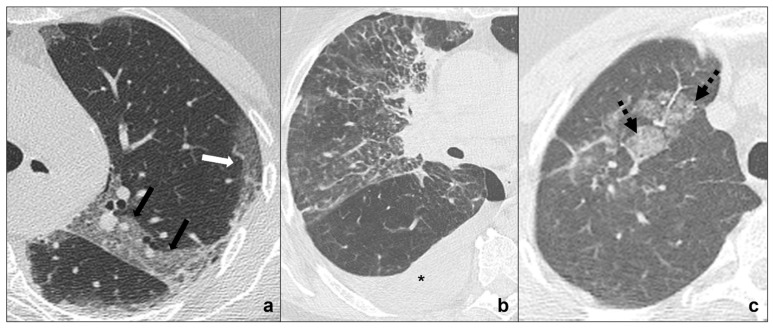
(**a**–**c**) HRTCs showing differential diagnoses of COVID-19 pneumonia stage 2/crazy-paving opacities. In stage 2, extensive, subpleural crazy-paving opacities (black arrows in (**a**)) coexist with subpleural areas of ground-glass (white arrow in (**a**)) and should be differentiated by lymphangitis carcinomatosis (**b**), which represents secondary spread of lung cancer to the lymphatic vessels and is frequently seen together with pleural effusion (black asterisk in (**b**)), and by radiation pneumonitis (acute radiation-induced lung disease) presenting with crazy-paving pattern (dotted black arrows in (**c**)) in the field of radiation. Lungs: mean window with 1500 HU; mean window level −600. Mediastinum: mean window with 350 HU; mean window level 50 HU.

It consists in tumor spreading through the lymphatics of the lung and it is most commonly secondary to adenocarcinoma [40]. Anamnesis and comparison with previous exams are suggested [41] (Figure 3b).

Radiological features for differential diagnosis between lymphangitis carcinomatosis and COVID-19 pneumonia [40,42] (Figure 3a,b):

1. evidence of coexisting solid/subsolid cancer;
2. unilateral involvement, which is homolateral to lung cancer;
3. crazy paving pattern is typical, but does not coexist, follow, or precede
4. ground-glass opacities and consolidations, that are typical, respectively, of stage one and three of COVID-19 pneumonia;
5. pleural effusions;
6. mediastinal lymphadenomegalies.

### 3.3. Coexistence of Ground-Glass or Crazy Paving with Consolidations

#### 3.3.1. MIA (Minimally Invasive Adenocarcinoma)

**Figure 4:**
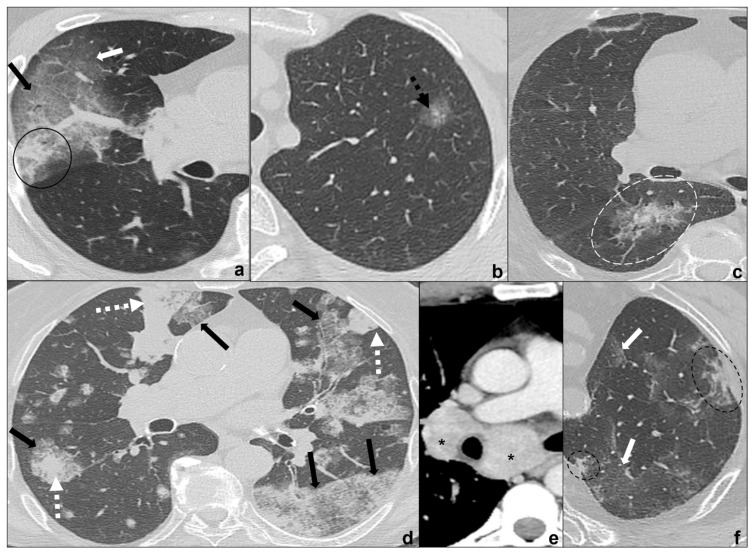
(**a**–**f**) HRTCs showing differential diagnoses of COVID-19 pneumonia stage 2–3/consolidations with ground-glass/crazy paving opacities. The transition between stage 2 and 3 of COVID-19 pneumonia is characterized by confluent consolidative foci (black circle in (**a**)) in extensive areas of ground-glass (white arrow in (**a**)) and/or crazy paving opacities (black arrow in (**a**)). Differential diagnosis with adenocarcinoma should include: minimally invasive adenocarcinoma presenting as a ground-glass area surrounding a solid nodule < 5 mm (dotted black arrow in (**b**)); invasive non-mucinous adenocarcinoma in which the nodule is >5mm and there is a small surrounding area of ground glass (dotted white circle in (**c**)); and invasive mucinous carcinoma, in particular the multicentric carcinoma (**d**) which presents as bilateral intraparenchymal or subpleural consolidations (dotted white arrows in (**d**)) surrounded by areas of ground-glass/crazy paving (black arrows in (**d**)) co-existing with extensive areas of ground-glass and crazy paving opacities (black arrows in (**d**)). Additional findings such as lymphadenopathies (black asterisks in (**e**)) may guide differential diagnosis. In (**f**), HRTC of patients presenting with chemotherapy induced organizing pneumonia characterized by extensive areas of ground-glass opacities (white arrows in (**f**)) coexisting with ill-defined consolidations (dotted black circle). Lungs: mean window with 1500 HU; mean window level −600. Mediastinum: mean window with 350 HU; mean window level 50 HU.

It is a minimally-invasive lepidic-growing tumor that may be seen as a focal ground-glass opacity surrounding a nodule ≤5 mm on CT scan [43,44]. The presence of focus or foci of invasive adenocarcinoma may help distinguish MIA from AIS [36], but the diagnosis can only be made after a complete histologic review [34,36] (Figure 4b).

#### 3.3.2. INMA (Invasive Non-Mucinous Adenocarcinoma)

Among the variants there is lepidic adenocarcinoma, which appears as a solid nodule (>5 mm) with a small surrounding ground glass opacity indicating lepidic growing on CT [26,38]. The size of focus of the invasive carcinoma being >5 mm has been shown to be an effective way to distinguish INMA from AIS and MIA [45] (Figure 4c).

#### 3.3.3. IMA (Invasive Mucinous Adenocarcinoma)

This is a focal or multicentric invasive lepidic-growing mucinous adenocarcinoma with a variety of different presentations from solid to non-solid on CT scan depending on mucin production [43,44] (Figure 4d).

Clinics and anamnesis are extremely important because tumors are often asymptomatic in the early phases or may show long-lasting symptoms. Radiological features for differential diagnosis of MIA, INMA, and IMA with COVID-19 pneumonia [18,34,36,46] (Figure 4a–e) include:

1. tumors are generally focal entities and appear as ground-glass opacities (atypical adenomatous hyperplasia likely being ≤5 mm, adenocarcinoma in situ generally being bigger, even measuring ≤3 cm), or ground-glass opacity surrounding a nodule (≤5 mm in minimally invasive adenocarcinoma and >5 mm in lepidic predominant adenocarcinoma);
2. even if tumors are multicentric, such as invasive mucinous adenocarcinoma, they tend to grow even after antibiotic or anti-inflammatory treatment;
3. additional findings such as cysts (cystadenocarcinoma);
4. cleavage invasion;
5. lymphangitic carcinomatosis (Figure 3b);
6. mediastinal lymphadenopathies (Figure 4e) and pleural effusions.

### 3.4. Consolidations

#### 3.4.1. Single Consolidation

##### Lung Tumor

Lung tumors may frequently appear as a single solid (<3 cm) or mass (>3 cm) with different morphological, immunohistochemical, and molecular characteristics which guide the differential diagnosis and treatment [18].

Imaging ensures an optimal evaluation of morphological features and histopathology is frequently mandatory for a definite differential diagnosis which will guide treatment. Differential diagnosis among lung cancer subtypes is beyond the scope of this article; therefore, we will focus on the typical presentation of single solid pulmonary lung cancer and of differential diagnosis with COVID-19 pneumonia phase 3.

**Figure 5:**
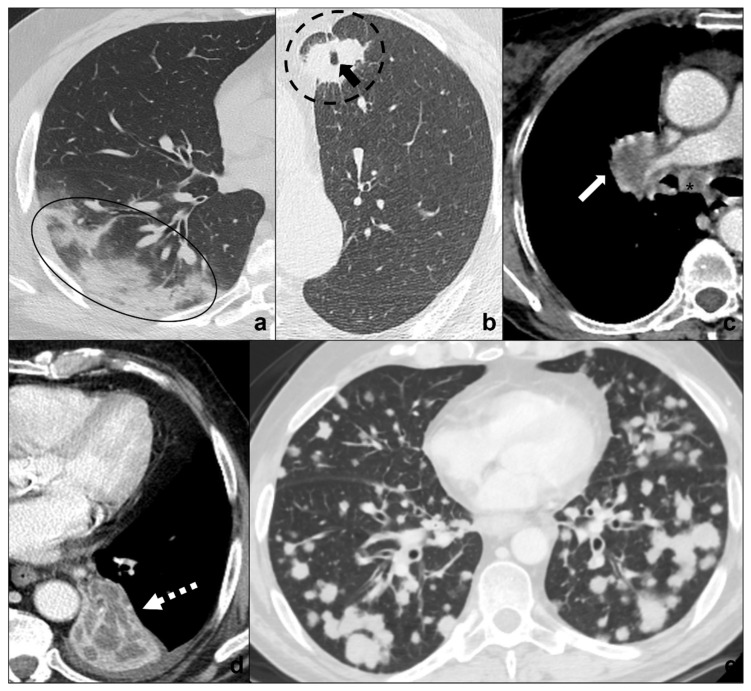
(**a**–**e**) Chest CTs showing differential diagnoses of COVID-19 pneumonia stage 3/consolidations. Subpleural ill-defined consolidations are typical of COVID-19 pneumonia stage 3 (black circle) and are generally easy distinguishable from lung cancer, especially if lung tumors present as big masses with spiculated margins, pleural retraction striae (dotted black circle in (**b**)), cavitations (black arrow in (**b**)) and lymphadenopathies (black asterisk in (**c**)). Contrast administration is useful to appreciate the inhomogeneous enhancement of lung tumor and mediastinal tissue infiltration (white arrow in (**c**)), and/or additional findings such as lobar atelectasis (dotted white arrow in (**d**)) secondary to bronchial invasion with progressive lobar hypoventilation and secretive stagnation. Solid metastases (**e**) may be differentiated by COVID-19 consolidations because they appear as roundish nodules with regular margins and a random distribution in relation to hematogenous spread. Lungs: mean window with 1500 HU; mean window level −600. Mediastinum: mean window with 350 HU; mean window level 50 HU.

Anamnesis and clinical signs and symptoms are crucial for the suspect of solid lung tumors. Radiological features for differential diagnosis of a single consolidation and COVID-19 pneumonia [18,34,46,47,48] (Figure 5a–d):

1. generally focal entities, appearing as a single nodule (<3 cm) or mass (>3 cm) with invasive and infiltrative features;
2. spiculated margins with pleural and parenchymal retraction stripes causing extensive pulmonary distortions and mediastinal attraction;
3. inhomogeneous density in relation to hemorrhagic and/or necrotic foci (e.g., small cell carcinoma, large cell carcinoma);
4. inhomogeneous contrast enhancement that is typical of cancer and is not seen in COVID-19 consolidations;
5. possible endobronchial growth and spread through airspace (STAS), which has been recognized as a feature with prognostic significance in 2021 WHO classification of thoracic tumors;
6. not generally associated with ground-glass or crazy paving areas. If ground-glass or crazy paving areas are present due to edema and hemorrhage, they do not follow COVID-19 pneumonia phases;
7. may be central or peripheral, but do not present a strictly subpleural distribution (squamous cell carcinoma and small cell carcinoma being generally central tumors whilst large cell carcinoma being often peripheral);
8. grow during antibiotics/anti-inflammatory therapies;
9. cysts and/or cavitations (squamous cell carcinoma);
10. chest wall, mediastinum and mediastinal organs invasion (frequent in small cell carcinoma, which is most common oncological cause of superior vena cava compressive/infiltrative/thrombotic obstruction);
11. associated findings such as atelectasis (Figure 5d) and post-obstructive pneumonia (typical of endobronchial growing tumors such as squamous cell carcinoma;
12. lymphangitis carcinomatosis;
13. mediastinal lymphadenopathies and pleural effusions;
14. systemic metastasis, which are particularly early and frequent in small cell carcinoma.

#### 3.4.2. Multiple Consolidations

##### Metastases

Metastases are multiple parenchymal and/or pleural lesions secondary to a primitive cancer. Solid metastases present as solid, homogenous nodules with random distribution, regular margins, and nodular morphology, even if there are rarer and atypical presentations [37,38,49].

Anamnesis and clinical signs and symptoms are crucial for the suspicion of solid lung tumors. Radiological features for differential diagnosis of multiple consolidations and COVID-19 pneumonia [38,39,49] (Figure 5a,e):

1. generally present as discrete regular nodules with roundish morphology while COVID-19 consolidations present ill-defined margins and do not show a nodular appearance;
2. generally present a random distribution in case of hematogenous spreading or may present a spreading through airspace (STAS);
3. may be asymptomatic and increase in number and dimensions during antibiotic and anti-inflammatory treatment;
4. are not frequently associated with ground-glass or crazy paving opacities and do not follow COVID-19 pneumonia phases.

## 4. COVID-19 Pneumonia and Treatment-Induced Lung Disease: Differential Diagnosis

Lung cancer therapy has been continuously evolving and is strictly dependent on neoplasm morphostructural, immunohistochemical, and molecular features [18]. On the other hand, oncological treatment is the cause of multiple acute and chronic lung diseases [27,50,51,52,53,54]. Anamnesis and timing of clinical signs and symptoms is pivotal for an optimal differential diagnosis and proper treatment [50].

### 4.1. RILD (Radiation-Induced Lung Disease)

**Figure 6:**
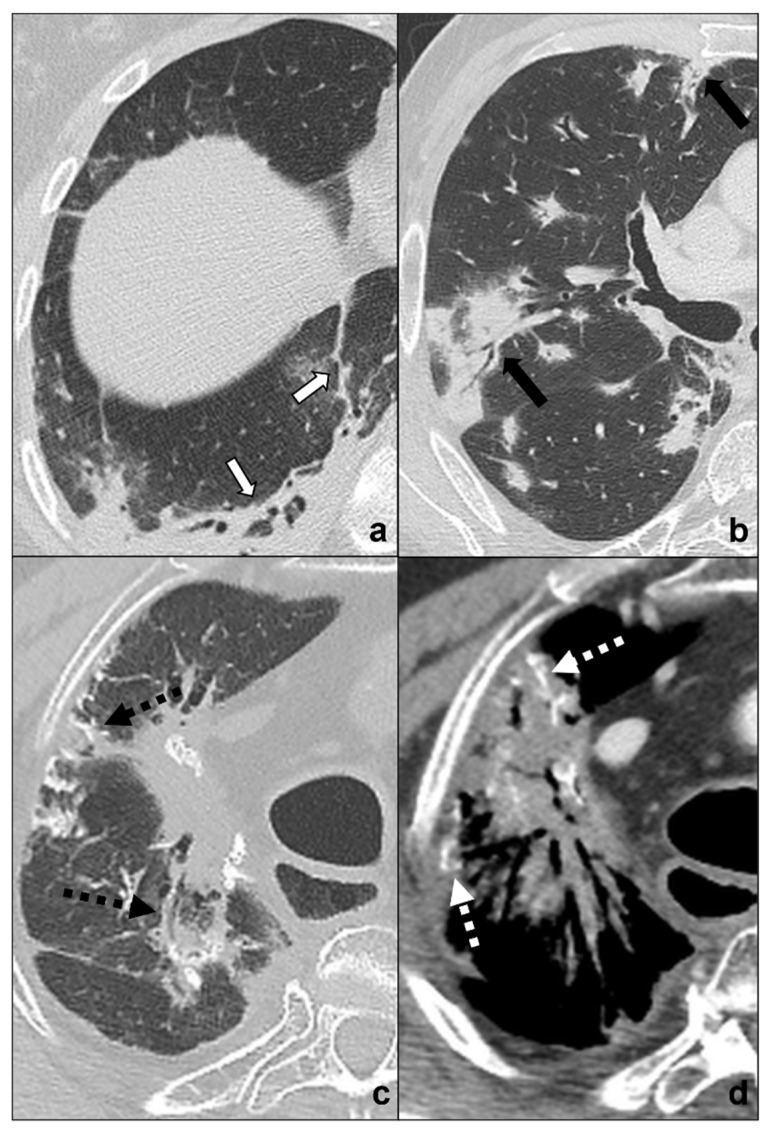
(**a**–**d**) Chest CTs showing differential diagnoses of COVID-19 pneumonia stage 4/absorption phase characterized by parenchymal bands (black-bordered white arrows in (**a**). Differential diagnoses generally include chemotherapy-induced lung disease (**b**) and radiation-induced lung disease (**c**,**d**). In (**b**), HRTC of a patient presenting with OP showing pulmonary parenchymal ill-defined bands and consolidations during a chemotherapy cycle (black arrows in (**b**)); in (**c**,**d**), chest CT of a patient who underwent radiotherapy for an apical lung cancer, showing an extensive mass with irregular margins, pleural and mediastinal retraction striae (black dotted arrow) and with some calcification within (dotted white arrows) in the field of radiation. Lungs: mean window with 1500 HU; mean window level −600. Mediastinum: mean window with 350 HU; mean window level 50 HU.

This is common after therapeutic irradiation of intrathoracic and chest wall malignancies [52] and can be divided into acute RILD (≤6 months from completion of therapy) (Figure 2c and Figure 3c) and chronic RILD (up to 2 years from completion of therapy) [51,53] (Figure 6c,d).

#### 4.1.1. Ground Glass and Crazy Paving

Acute RILD often presents as areas of ground-glass opacities (Figure 2c) which may overlap with interstitial thickening resulting in a crazy paving pattern (Figure 3c). The densitometric alterations strictly reflect radiation fields [53].

#### 4.1.2. Consolidations and Absorption Phase

Radiations may induce abnormal pulmonary reaction resulting in linear fibrotic parenchymal bands to massive consolidations causing retraction on pleural/parenchymal tissue and mediastinum, bronchiectasis, and architectural distortions. These areas are generally located in the radiation field boundaries [53].

Anamnesis of radiation exposure and comparison with previous CT scans are necessary for a correct differential diagnosis. Radiological features for differential diagnosis between RILD and COVID-19 pneumonia [51,53]:

1. parenchymal lesions located within radiation field boundaries, in particular ground-glass or crazy-paving opacities in acute RILD and consolidations with calcification foci in chronic RILD;
2. additional findings such as nodules, atelectasis, and tree-in-bud, more common in radiation pneumonitis;
3. evidence of fibrosis, traction bronchiectases, volume loss, architectural distortion, and ipsilateral displacement of mediastinum during the chronic stage;
4. pleural effusions in the early stage and pleural thickening in the chronic stage.

### 4.2. Chemotherapy- and Immunotherapy-Induced Lung Disease

We will cite and classify the most common chemotherapy and immunotherapy-induced pulmonary diseases in relation to COVID-19 pneumonia phases [27,50,54]:

#### 4.2.1. Ground-Glass and Crazy Paving

NSIP (non-specific interstitial pneumonia): methotrexate, carmustine, chlorambucil;

Hypersensitivity pneumonitis: immunosuppressants such as sirolimus and everolimus;

Pulmonary hemorrhage: cytarabine, cyclophosphamide.

OP (organizing pneumonia): Bleomycin, methotrexate, cyclophosphamide (Figure 4f).

#### 4.2.2. Consolidations

Pulmonary hemorrhage: cytarabine, cyclophosphamide;

OP (organizing pneumonia): bleomycin, methotrexate, cyclophosphamide (Figure 4f and Figure 6b).

#### 4.2.3. Absorption Phase

OP (organizing pneumonia): bleomycin, methotrexate, cyclophosphamide (Figure 6b).

#### 4.2.4. ARDS

ARDS/DAD (diffuse alveolar damage): bleomycin, carmustine, cyclophosphamide, mitomycin, melphalan.

## 5. COVID-19 Pneumonia and Lung Cancer: Overlapping Pathologies

Cancer patients are more prone to infections, and in particular to COVID-19 infection, and often present more severe forms of COVID-19 pneumonia leading to dire complications [11,12,13,14].

**Figure 7:**
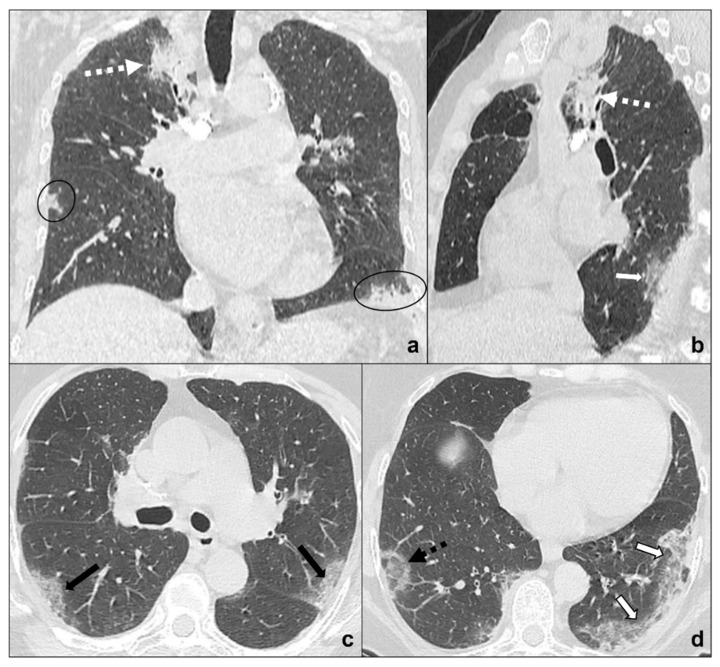
(**a**–**d**) Chest CT of an oncologic patient affected by a paramediastinal lung cancer located in the apical segment of the right upper lobe (dotted white arrow) and undergoing chemotherapy and radiotherapy. After admission to the first aid with ageusia, anosmia, and fever, a chest CT scan was performed showing subpleural areas of ground-glass (white arrow in (**b**)) and crazy paving (black arrow in (**c**)) with the typical reversed halo sign or atoll sign (dotted black arrow in (**d**)), parenchymal consolidations (black circle in (**a**)) and subpleural parenchymal bands together with crazy paving opacities (black-bordered white arrow in (**d**)). Lungs: mean window with 1500 HU; mean window level −600. Mediastinum: mean window with 350 HU; mean window level 50 HU.

Although COVID-19 pneumonia may be an incidental diagnosis in asymptomatic patients [15], acute symptoms worsening and changing should always suggest an overlapping pathology or a progression and require urgent investigation (Figure 7a–d).

Radiologists play an important role since a chest CT scan is a non-invasive technique which may suggest a definite diagnosis.

Key radiological features for a differential diagnosis between lung cancer and treatment-induced lung disease have been extensively presented in the previous paragraphs. We will provide the clinical radiologist with some crucial tips and radiological elements for a proper diagnosis:

1. oncological patient CT protocol requires contrast administration for an optimal evaluation of lung cancer, lymphadenopathies, and mediastinal and vessel invasion. HRCT performed in COVID-19 patients should be followed by a contrast-enhanced CT scan in case of the suspicion of co-existing cancer;
2. radiological features should always be paired with a patient’s specific lung tumor because a discrepancy between known histology and radiological presentation may guide the diagnosis of overlapping pathologies;
3. compare current CT scan with previous exams to exclude cancer progression or regression, treatment-induced lung diseases, and overlapping of COVID-19 pneumonia;
4. search for typical phase progression of COVID-19 pneumonia in multiple CT scans and compare it with clinical symptoms;
5. identify possible regression or conversely progression after COVID-19 treatment in case of non-univocal radiological features;
6. always discuss the case in multidisciplinary meetings to have an optimal and wide knowledge of the patient’s anamnesis, signs and symptoms, and pathology and treatment.

Mild radiological findings in patients with chronic lung disease may represent a dire challenge for the radiologist in the pandemic era, since not many typical features or non-COVID extra findings may dissimulate diagnosis. If clinics, laboratory, and tampons do not help the diagnosis but the suspicion remains high, our suggestion is to repeat the CT scan, since in the first days tampons and CT may be negative and clinical and laboratory findings may be ambiguous [28]. The radiological elements we suggest for COVID-19 and lung cancer differential diagnosis may be used to develop AI protocols for the identification of subtle radiological features of COVID-19 pneumonia to guarantee a proper treatment [6]. Further studies are required to address this issue.

## 6. Conclusions

COVID-19 pneumonia has been monopolizing health systems’ economic and human resources world-wide with consequences for cancer diagnosis and treatment, such as the late diagnosis of lung cancer leading to patients’ poor prognosis and late medical treatment [3,4]. Oncological patients are more prone to present severe forms of COVID-19 pneumonia and are subject to worse complications [5,6,7]. Radiologists have the challenging role of suggesting a differential diagnosis with lung cancer and treatment-induced lung diseases or suggesting an overlap between these pathologies. Despite similarities or overlapping findings, the combination of clinics and some specific radiological findings, which are also identified by comparison with previous and follow-up CT scans, may guide differential diagnosis. It is crucial to search for typical COVID-19 pneumonia phase progression and typical radiological features on HRTC. The evidence of atypical findings, such as lymphadenopaties and mediastinal and vessel invasion, as well as the absence of response to therapy, should arouse the suspicion of lung cancer and require contrast administration. Ground-glass areas and/or consolidations bound to radiotherapy fields or pneumonitis arising during and after oncological therapy should always arouse the suspicion of radiation-induced lung disease and chemo/immunotherapy-induced lung disease. The radiological elements we suggested for COVID-19 and lung cancer differential diagnosis may be used to develop AI protocols to guarantee an early and proper diagnosis and treatment to improve patients’ quality of life and life expectancy.

## Figures and Tables

**Table 1:** COVID-19 pneumonia main radiological features, additional findings, and spatial distribution [19]. The table was reproduced from Guarnera, A.; Podda, P.; Santini, E.; Paolantonio, P.; Laghi, A. Differential diagnoses of COVID-19 pneumonia: the current challenge for the radiologist-a pictorial essay. *Insights Imaging* **2021**, *12*, 34 [32], under the terms and conditions of the Creative Commons Attribution (CC BY) license (http://creativecommons.org/licenses/by/4.0/) (accessed on 26 December 2021).

Stage	Phase	Timing (days)	Predominant Radiological Findings	Additional Findings	Spatial Distribution of Radiological Findings
1	Early	0–4	Ground glass opacities	Peripheral vessel widening Halo sign Atoll sign or reversed halo sign Overlapping of radiological findings in different phases Rarity of: lymphadenopathies, pleural effusions, pulmonary nodules	Bilateral Peripheral/subpleural Centro-parenchymal (atypical) Lower lobes prevalence
2	Progressive	5–8	Crazy paving pattern, ground glass opacities and small consolidations		
3	Peak	9–13	Consolidative foci		
4	Absorption	≥14	Ground-glass opacities and linear consolidation		

## Data Availability

The data are available from the corresponding author, A.G., upon reasonable request.

## Abbreviations

SARS-CoV-2: Severe Acute Respiratory Syndrome Coronavirus-2; CT: computed tomography; HRCT: high resolution computed tomography; WHO: world health organization; ARDS: acute respiratory distress syndrome; AIS: adenocarcinoma in situ; AAH: atypical adenomatous hyper-plasia, MIA: minimally invasive adenocarcinoma; INMA: invasive non-mucinous adenocarci-noma; IMA: invasive mucinous adenocarcinoma; STAS: Spread thought airspace; RILD: radia-tion-induced lung disease; NSIP: non-specific interstitial pneumonia; OP: organizing pneumonia; DAD: diffuse alveolar damage; AI: artificial intelligence.

## References

[1] Chan J.F.-W., Yuan S., Kok K.-H., To K.K.-W., Chu H., Yang J., Xing F., Liu J., Yip C.C.-Y., Poon R.W.-S. (2020). A Familial Cluster of Pneumonia Associated with the 2019 Novel Coronavirus Indicating Person-to-Person Transmission: A Study of a Family Cluster. Lancet.

[2] Tracking SARS-CoV-2 Variants. https://www.who.int/activities/tracking-SARS-CoV-2-variants.

[3] Classification of Omicron (B.1.1.529): SARS-CoV-2 Variant of Concern. https://www.who.int/news/item/26-11-2021-classification-of-omicron-(b.1.1.529)-SARS-CoV-2-variant-of-concern.

[4] Talic S., Shah S., Wild H., Gasevic D., Maharaj A., Ademi Z., Li X., Xu W., Mesa-Eguiagaray I., Rostron J. (2021). Effectiveness of Public Health Measures in Reducing the Incidence of COVID-19, SARS-CoV-2 Transmission, and COVID-19 Mortality: Systematic Review and Meta-Analysis. BMJ.

[5] Mitrev L.V., Banerjee A., Van Helmond N. (2021). Correlation of Country Characteristics and Government Response Measures with COVID-19 Mortality during the First Phase of the Global COVID-19 Pandemic: A Worldwide Ecological Study. Cureus.

[6] Fusco R., Grassi R., Granata V., Setola S.V., Grassi F., Cozzi D., Pecori B., Izzo F., Petrillo A. (2021). Artificial Intelligence and COVID-19 Using Chest CT Scan and Chest X-Ray Images: Machine Learning and Deep Learning Approaches for Diagnosis and Treatment. J. Pers. Med..

[7] Alruwaili M., Shehab A., Abd El-Ghany S. (2021). COVID-19 Diagnosis Using an Enhanced Inception-ResNetV2 Deep Learning Model in CXR Images. J. Healthc. Eng..

[8] Dey S., Bhattacharya R., Malakar S., Mirjalili S., Sarkar R. (2021). Choquet Fuzzy Integral-Based Classifier Ensemble Technique for COVID-19 Detection. Comput. Biol. Med..

[9] Englum B.R., Prasad N.K., Lake R.E., Mayorga-Carlin M., Turner D.J., Siddiqui T., Sorkin J.D., Lal B.K. (2021). Impact of the COVID-19 Pandemic on Diagnosis of New Cancers: A National Multicenter Study of the Veterans Affairs Healthcare System. Cancer.

[10] Dinmohamed A.G., Visser O., Verhoeven R.H.A., Louwman M.W.J., van Nederveen F.H., Willems S.M., Merkx M.A.W., Lemmens V.E.P.P., Nagtegaal I.D., Siesling S. (2020). Fewer Cancer Diagnoses during the COVID-19 Epidemic in the Netherlands. Lancet Oncol..

[11] Kamboj M., Sepkowitz K.A. (2009). Nosocomial Infections in Patients with Cancer. Lancet Oncol..

[12] Kuderer N.M., Choueiri T.K., Shah D.P., Shyr Y., Rubinstein S.M., Rivera D.R., Shete S., Hsu C.-Y., Desai A., de Lima Lopes G. (2020). Clinical Impact of COVID-19 on Patients with Cancer (CCC19): A Cohort Study. Lancet.

[13] Zhang H., Han H., He T., Labbe K.E., Hernandez A.V., Chen H., Velcheti V., Stebbing J., Wong K.-K. (2021). Clinical Characteristics and Outcomes of COVID-19-Infected Cancer Patients: A Systematic Review and Meta-Analysis. J. Natl. Cancer Inst..

[14] Liu C., Zhao Y., Okwan-Duodu D., Basho R., Cui X. (2020). COVID-19 in Cancer Patients: Risk, Clinical Features, and Management. Cancer Biol. Med..

[15] Yoo K., Choi R.Y., Sun J., Veselis C., Kamat B., Kumaran M., Agosto O., Maresky H.S. (2020). Incidental COVID-19 in the Radiology Department: Radiographic Findings of COVID-19 in Asymptomatic Patient Undergoing CT Staging for Breast Cancer. Radiol. Case Rep..

[16] Catania C., Stati V., Spitaleri G. (2020). Interstitial Pneumonitis in the COVID-19 Era: A Difficult Differential Diagnosis in Patients with Lung Cancer. Tumori J..

[17] Dai M., Liu D., Liu M., Zhou F., Li G., Chen Z., Zhang Z., You H., Wu M., Zheng Q. (2020). Patients with Cancer Appear More Vulnerable to SARS-CoV-2: A Multi-Center Study during the COVID-19 Outbreak. Cancer Discov..

[18] WHO (2021). Classification WHO Classification of Tumours Editorial Board Thoracic Tumours.

[19] Zhou P., Yang X.-L., Wang X.-G., Hu B., Zhang L., Zhang W., Si H.-R., Zhu Y., Li B., Huang C.-L. (2020). A Pneumonia Outbreak Associated with a New Coronavirus of Probable Bat Origin. Nature.

[20] Thachil J. (2020). The Versatile Heparin in COVID-19. J. Thromb. Haemost..

[21] Tang N., Bai H., Chen X., Gong J., Li D., Sun Z. (2020). Anticoagulant Treatment Is Associated with Decreased Mortality in Severe Coronavirus Disease 2019 Patients with Coagulopathy. J. Thromb. Haemost..

[22] Batta Y., King C., Johnson J., Haddad N., Boueri M., Haddad G. (2021). Sequelae and Comorbidities of COVID-19 Manifestations on the Cardiac and the Vascular Systems. Front. Physiol..

[23] Rubin G.D., Ryerson C.J., Haramati L.B., Sverzellati N., Kanne J.P., Raoof S., Schluger N.W., Volpi A., Yim J.-J., Martin I.B.K. (2020). The Role of Chest Imaging in Patient Management during the COVID-19 Pandemic: A Multinational Consensus Statement from the Fleischner Society. Radiology.

[24] Wong H.Y.F., Lam H.Y.S., Fong A.H.-T., Leung S.T., Chin T.W.-Y., Lo C.S.Y., Lui M.M.-S., Lee J.C.Y., Chiu K.W.-H., Chung T.W.-H. (2020). Frequency and Distribution of Chest Radiographic Findings in Patients Positive for COVID-19. Radiology.

[25] Jacobi A., Chung M., Bernheim A., Eber C. (2020). Portable Chest X-Ray in Coronavirus Disease-19 (COVID-19): A Pictorial Review. Clin. Imaging.

[26] Pan F., Ye T., Sun P., Gui S., Liang B., Li L., Zheng D., Wang J., Hesketh R.L., Yang L. (2020). Time Course of Lung Changes at Chest CT during Recovery from Coronavirus Disease 2019 (COVID-19). Radiology.

[27] Guarnera A., Santini E., Podda P. (2021). Idiopathic Interstitial Pneumonias and COVID-19 Pneumonia: Review of the Main Radiological Features and Differential Diagnosis. Tomography.

[28] Bernheim A., Mei X., Huang M., Yang Y., Fayad Z.A., Zhang N., Diao K., Lin B., Zhu X., Li K. (2020). Chest CT Findings in Coronavirus Disease-19 (COVID-19): Relationship to Duration of Infection. Radiology.

[29] Hansell D.M., Bankier A.A., MacMahon H., McLoud T.C., Müller N.L., Remy J. (2008). Fleischner Society: Glossary of Terms for Thoracic Imaging. Radiology.

[30] Kanne J.P., Little B.P., Chung J.H., Elicker B.M., Ketai L.H. (2020). Essentials for Radiologists on COVID-19: An Update—Radiology Scientific Expert Panel. Radiology.

[31] Sheard S., Rao P., Devaraj A. (2012). Imaging of Acute Respiratory Distress Syndrome. Respir. Care.

[32] Guarnera A., Podda P., Santini E., Paolantonio P., Laghi A. (2021). Differential Diagnoses of COVID-19 Pneumonia: The Current Challenge for the Radiologist-a Pictorial Essay. Insights Imaging.

[33] Travis W.D., Brambilla E., Burke A., Nicholson A.G. (2015). International Agency for Research on Cancer. WHO Classification of Tumours of the Lung, Pleura, Thymus and Heart.

[34] Travis W.D., Brambilla E., Nicholson A.G., Yatabe Y., Austin J.H.M., Beasley M.B., Chirieac L.R., Dacic S., Duhig E., Flieder D.B. (2015). The 2015 World Health Organization Classification of Lung Tumors: Impact of Genetic, Clinical and Radiologic Advances since the 2004 Classification. J. Thorac. Oncol..

[35] Tang E.R., Schreiner A.M., Pua B.B. (2014). Advances in Lung Adenocarcinoma Classification: A Summary of the New International Multidisciplinary Classification System (IASLC/ATS/ERS). J. Thorac. Dis..

[36] Hutchinson B.D., Shroff G.S., Truong M.T., Ko J.P. (2019). Spectrum of Lung Adenocarcinoma. Semin. Ultrasound CT MR.

[37] Nishi W., Hayashi K. (2021). [Lung Metastases from Pancreatic Cancer Initially Suspected to be Primary Lung Cancer due to Lepidic Tumor Growth]. Kyobu Geka.

[38] Seo J.B., Im J.G., Goo J.M., Chung M.J., Kim M.Y. (2001). Atypical Pulmonary Metastases: Spectrum of Radiologic Findings. Radiographics.

[39] Gagnon M.-H., Wallace A.B., Yedururi S., Khanna G. (2021). Atypical Pulmonary Metastases in Children: Pictorial Review of Imaging Patterns. Pediatr. Radiol..

[40] Zhuang L., Liu X., Hu C., Zhang L., Jiang G., Wu J., Zheng S. (2014). Pulmonary Lymphangitic Carcinomatosis in Liver Carcinoma: A Rare Case Report and Literature Review. World J. Surg. Oncol..

[41] Wallach J.B., McGarry T., Torres J. (2011). Lymphangitic Metastasis of Recurrent Renal Cell Carcinoma to the Contralateral Lung Causing Lymphangitic Carcinomatosis and Respiratory Symptoms. Curr. Oncol..

[42] Biswas A., Sriram P.S. (2015). Getting the Whole Picture: Lymphangitic Carcinomatosis. Am. J. Med..

[43] Austin J.H.M., Garg K., Aberle D., Yankelevitz D., Kuriyama K., Lee H.-J., Brambilla E., Travis W.D. (2013). Radiologic Implications of the 2011 Classification of Adenocarcinoma of the Lung. Radiology.

[44] Travis W.D., Brambilla E., Noguchi M., Nicholson A.G., Geisinger K.R., Yatabe Y., Beer D.G., Powell C.A., Riely G.J., Van Schil P.E. (2011). International Association for the Study of Lung Cancer/american Thoracic Society/european Respiratory Society International Multidisciplinary Classification of Lung Adenocarcinoma. J. Thorac. Oncol..

[45] MacMahon H., Naidich D.P., Goo J.M., Lee K.S., Leung A.N.C., Mayo J.R., Mehta A.C., Ohno Y., Powell C.A., Prokop M. (2017). Guidelines for Management of Incidental Pulmonary Nodules Detected on CT Images: From the Fleischner Society 2017. Radiology.

[46] Nicholson A.G., Tsao M.S., Beasley M.B., Borczuk A.C., Brambilla E., Cooper W.A., Dacic S., Jain D., Kerr K.M., Lantuejoul S. (2021). The 2021 WHO Classification of Lung Tumors: Impact of Advances since 2015. J. Thorac. Oncol..

[47] Carter B.W., Glisson B.S., Truong M.T., Erasmus J.J. (2014). Small Cell Lung Carcinoma: Staging, Imaging, and Treatment Considerations. Radiographics.

[48] Collins J. (2015). Med Chest Radiology: The Essentials.

[49] Martínez-Jiménez S., Rosado-de-Christenson M.L., Walker C.M., Kunin J.R., Betancourt S.L., Shoup B.L., Pettavel P.P. (2014). Imaging Features of Thoracic Metastases from Gynecologic Neoplasms. Radiographics.

[50] Rossi S.E., Erasmus J.J., McAdams H.P., Sporn T.A., Goodman P.C. (2000). Pulmonary Drug Toxicity: Radiologic and Pathologic Manifestations. Radiographics.

[51] Davis S.D., Yankelevitz D.F., Henschke C.I. (1992). Radiation Effects on the Lung: Clinical Features, Pathology, and Imaging Findings. Am. J. Roentgenol..

[52] Choi Y.W., Munden R.F., Erasmus J.J., Park K.J., Chung W.K., Jeon S.C., Park C.-K. (2004). Effects of Radiation Therapy on the Lung: Radiologic Appearances and Differential Diagnosis. Radiographics.

[53] Benveniste M.F.K., Welsh J., Godoy M.C.B., Betancourt S.L., Mawlawi O.R., Munden R.F. (2013). New Era of Radiotherapy: An Update in Radiation-Induced Lung Disease. Clin. Radiol..

[54] Sridhar S., Kanne J.P., Henry T.S., Revels J.W., Gotway M.B., Ketai L.H. (2021). Medication-Induced Pulmonary Injury: A Scenario- and Pattern-Based Approach to a Perplexing Problem. Radiographics.

